# Diagnosis and Treatment of Ankylosing Spondylitis

**DOI:** 10.7759/cureus.52559

**Published:** 2024-01-19

**Authors:** Parv Agrawal, Sachin Tote, Bhagyesh Sapkale

**Affiliations:** 1 Medicine, Jawaharlal Nehru Medical College, Datta Meghe Institute of Higher Education and Research, Wardha, IND; 2 Anatomy, Jawaharlal Nehru Medical College, Datta Meghe Institute of Higher Education and Research, Wardha, IND

**Keywords:** biologic treatments, axial skeleton, hla-b27, spondyloarthritis, ankylosing spondylitis

## Abstract

Ankylosing spondylitis (AS) is a chronic inflammatory condition primarily affecting the axial bone and sacroiliac joints. Its etiology is complicated and involves genetic variables, demographic factors (age of onset, gender, ethnicity, family history), and environmental variables. It typically manifests in males in their third decade. Galen is credited with first recognizing it, according to historical traditions, but it was not until the 19th century that specific diagnostic criteria were developed. The human leukocyte antigen B27 (HLA-B27) variation, around 20% of the genetic risk, is currently the most significant gene associated with AS susceptibility. Over 100 genes have been connected to AS susceptibility. Clinical signs of AS include stiffness and inflammation in the back, eye inflammation, aortitis (inflammation of the aorta), and spinal ankylosis that impacts posture and fatigue. The dagger sign and sacroiliitis on radiographs, in particular, are crucial for diagnosis. Early inflammatory alterations can be found using modern diagnostic tools such as MRI, and the HLA-B27 gene can help confirm the diagnosis. Overall, 80-95% of people with AS have the HLA-B27 marker.

Furthermore, although non-specific, elevated inflammatory markers, such as C-reactive protein and erythrocyte sedimentation rate, offer supporting evidence. Over time, treatment paradigms have seen significant change. First-line treatments such as non-steroidal anti-inflammatory drugs are no longer the only options, even though disease-modifying anti-rheumatic drugs and biologics, especially tumor necrosis factor blockers, have been developed. Physical therapy, which emphasizes consistent exercise, stretches, and posture maintenance, is extremely helpful in managing AS. Surgical interventions can be required in extreme situations. The significance of the interleukin 23/17 axis in the disease cascade has been demonstrated by recent research. Furthermore, a deeper comprehension of the genetic landscape, mainly the functions of non-HLA-B27 loci, may open the door for more specialized therapies. Early diagnosis and interdisciplinary therapies can improve patient outcomes and quality of life as our understanding of AS grows.

## Introduction and background

Ankylosing spondylitis (AS) is a kind of spondyloarthritis (SpA) characterized by chronic immune-mediated inflammatory arthritis. The axial skeleton and the sacroiliac joints are primarily affected, and it is more frequent in men in their third decade of life [1]. Although Galen provided the earliest accounts, it was not until the 19th century that reports from Pierre Marie, Adolph Strümpell, and Vladimir Bekhterev allowed for a precise diagnosis of the illness. While the human leukocyte antigen (HLA) B27 variant is known to be associated with the disease, other genes are involved in its onset. The era of biological treatments, which revolutionized the treatment and prognosis of AS, was ushered in by identifying multiple inflammatory pathways [2]. Although the mechanisms underpinning most relationships are unknown, it has been discovered that over 100 genes influence AS susceptibility [3]. The HLA-B27 marker is present in 80-95% of those with AS [1,2]. HLA-B57 in HIV, HLA-DRB1 in rheumatoid arthritis, HLA-DQ2/DQ8 in celiac disease, HLA-DR15 in multiple sclerosis, and HLA-A3/A11 in AS are additional significant HLAs that impact prognosis and clinical presentation in addition to HLA-B27. In addition to the substantial impact of HLA-B27, which determines around 20% of the hereditary risk, 113 established loci that contribute roughly 10% of the heritability of AS have been identified [4]. This represents significant progress in the identification of susceptibility alleles in the condition. Over the past few decades, the treatment of AS has been significantly improved by the use of biologics, tumor necrosis factor (TNF)-specific treatments, disease-modifying antirheumatic drugs (DMARDs), and non-steroidal anti-inflammatory drugs (NSAIDs) [5]. Illnesses, such as viral infection and illnesses with modified comorbidities, can dramatically increase the risk of AS [6]. AS can be triggered by specific bacterial infections, mainly gastrointestinal and respiratory infections; research on the association between AS and COVID-19 is underway. Despite evidence that interleukin (IL) 23 works as a precursor to Th17 cells, the T lymphocytes that produce IL-17, and even though IL-23 inhibition exhibits substantial efficacy in the treatment of psoriasis, blocking IL-23 did not demonstrate any clinical efficacy in the treatment of axial spondyloarthritis [7]. First, IL-23R pathway single-nucleotide polymorphisms are prevalent in all above-mentioned disorders, according to genome-wide association studies, even though not all of these illnesses are associated with HLA-B27 [8]. As a result, the IL-23 route serves as the standard genetic connection between all these disorders. Exercise is one of the many treatments for AS that have been suggested [9].

## Review

Search methodology

**Figure 1:**
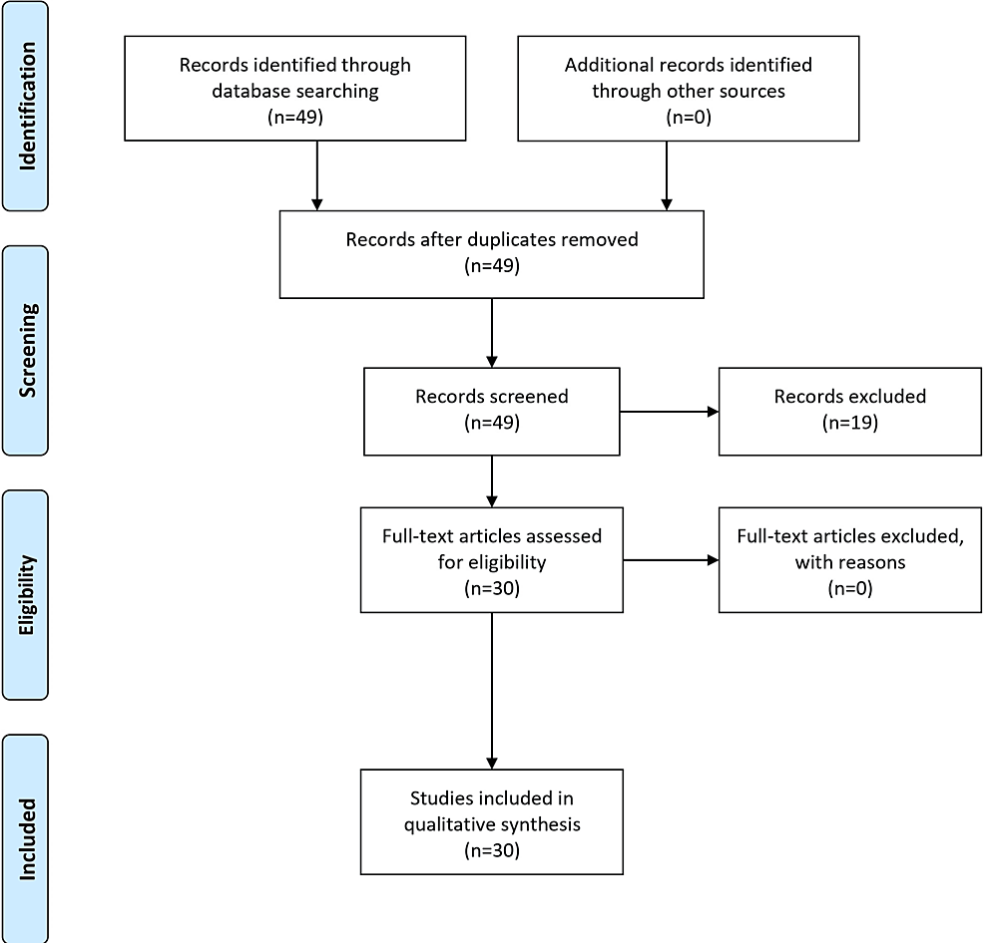
Preferred Reporting Items for Systematic Reviews and Meta-Analyses flow diagram.

A systemic review search was used to gain a thorough understanding of AS. The process was developed to ensure the most relevant studies from 2000 to 2022 were included. The central databases used for the search were Clevland Clinic, PubMed, and MayoClinic. To ensure the best outcomes, the following keywords were used in this study: “Ankylosing Spondylitis,” “spondyloarthritis,” “HLA-B27,” “axial skeleton,” “treatment,” “biologic treatments,” “diagnosis,” and “pathogenesis.” Combinations of terms such as “Ankylosing Spondylitis AND diagnosis” or “HLA-B27 AND treatment outcomes” were utilized to narrow the search further. This method enabled extracting specialized articles that concentrated on particular AS-related topics. The primary inclusion criteria were English language proficiency, peer-reviewed status, recent publication within the last 10 years, and relevance to AS. With exclusion criteria designed to preserve the information’s quality, relevance, and recentness, the resulting pool of 30 publications served as the basis for an extensive assessment. Subsequently, abstracts were scrutinized to weed out research that did not specifically address the fundamental elements of AS. A full-text review of the remaining papers was conducted to determine their applicability to the subject. Finally, the bibliographies of the chosen publications were manually searched to find any other research that might have been overlooked during the original search. This aided in finding books or earlier literature that gave background information about AS. Thirty articles comprised the final pool, which served as the foundation for the systematic review of AS. The Preferred Reporting Items for Systematic Reviews and Meta-Analyses flow diagram is shown in Figure 1.

Anatomical aspect of ankylosing spondylitis

The hips, knees, ankles, and shoulders are peripheral joints that could be affected. Inflammatory pain and stiffness in the back are common symptoms of AS, and the most recognizable symptoms are syndesmophytes and spinal ankylosis. Physical function and quality of life may be influenced by spinal ossification [10]. Controlling inflammation or disease activity may directly impact patient-reported outcomes and stop the further advancement of structural damage. Research into the function of the IL-23/17 axis in human diseases has become more apparent as the existence of innate and adaptive immune cell populations that can produce IL-17A in specific target organs may be linked to the suppression of IL-17A [11]. Despite comorbidities, resistance training is safe for the musculoskeletal and cardiovascular systems [12]. The use of these activities for therapeutic, rehabilitative, and health promotion objectives is now supported in AS by evidence. Advanced AS patients may have a variety of distinctive characteristics.

Patients frequently have a hunched-over posture with visible rigidity in the back [13]. Sacroiliac joint tenderness, which denotes inflammation, is frequently felt bilaterally. AS often manifests in people in their mid-20s and is more likely to occur in men [14]. A particular type of AS, called juvenile AS or juvenile-onset AS, can affect younger individuals [14]. The condition known as non-radiographic axial spondyloarthritis (nr-axSpA) usually first appears in the mid-20s and is more common in men. It may be associated with specific bacterial infections, mainly gastrointestinal and respiratory infections. It results in severe morbidity in both physical and mental health, which influences work productivity. In addition to uniting people with a typical disease pattern and allowing for advanced research, for individuals with psoriatic arthritis and inflammatory bowel illness associated with pre-radiographic AS and AS-like conditions, the axSpA umbrella provides earlier diagnosis and improved treatment [15].

Sacroiliitis spectrum: from mild discomfort to ankylosing spondylitis

From mild to severe and chronic stages, there are many different ways that sacroiliitis can present. In mild instances, imaging examinations show slight alterations and minor inflammation; people may only feel mild discomfort or pain [12]. More significant inflammation and pain are hallmarks of moderate sacroiliitis, frequently accompanied by imaging alterations showing abnormalities in the sacroiliac joint. Severe sacroiliitis, excruciating pain, stiffness, and noticeable joint changes, such as considerable constriction and bone erosions, are the hallmarks of severe sacroiliitis in patients [11]. Imaging evidence of joint fusion or ankylosis may be present, along with ongoing inflammation and recurring episodes of pain and stiffness, all indicative of chronic sacroiliitis. AS, a chronic inflammatory disease affecting the spine, is sometimes associated with sacroiliitis [10]. AS causes gradual spine stiffness and fusion, leading to significant motor and functional impairments [13]. Reduced influence on an individual’s well-being is achieved through early diagnosis and appropriate care of sacroiliitis. The diagnostic aspects of AS are shown in Table 1.

**Table 1:** Diagnosis of ankylosing spondylitis. HLA-B27: human leukocyte antigen B27; CRP: C-reactive protein; ESR: erythrocyte sedimentation rate

Aspect of diagnosis	Details
Diagnosis challenges	Early symptoms can overlap with other conditions
Supporting diagnostic evidence	Radiographic evidence
Primary diagnostic criteria	Clinical presentation
Hallmark radiographic feature	Sacroiliitis visible on X-rays
Genetic marker	Presence of the HLA-B27 gene (in 80–95% of AS individuals) (not exclusive to AS; found in a significant proportion of the general population)
Laboratory tests	Elevated inflammatory markers such as CRP and ESR (non-specific)
Diagnostic importance	A thorough clinical examination, detailed medical history, and exclusion of other potential causes are crucial.
MRI findings	When non-radiographic axial spondyloarthritis is present, MRI can detect early inflammatory changes that are not apparent on conventional radiographs. Specific lesions can also increase the disease's positive predictive value
MRI findings and lesions of AS	Erosions at entheses. Bone tissue loss due to inflammation. A strong indicator of active disease
	Sclerosis. Abnormal tissue hardening in sacroiliac joints and spine. Suggestive of chronic inflammation and structural changes
	Fatty lesions. Presence in areas of active inflammation. Supports the likelihood of AS diagnosis, especially when combined with other findings.
Degrees of sacroiliitis	Mild sacroiliitis: subtle imaging alterations and minimal inflammation
	Moderate sacroiliitis: pronounced inflammation and noticeable pain
	Severe sacroiliitis: excruciating pain, stiffness, and marked joint
	Changes chronic sacroiliitis: ongoing inflammation and recurring episodes
	Ankylosing spondylitis: association with sacroiliitis and progressive fusion

Treatment approaches for ankylosing spondylitis

According to the 2009 Assessment in Ankylosing Spondylitis (ASAS) classification criteria, the difference between radiographic and non-radiographic types of classical AS is whether or not the sacroiliac joints exhibit specific radiographic changes, also known as radiographic axSpA [16]. In patients with AS, NSAIDs relieve symptoms by reducing inflammation [17]. DMARDs may take weeks or months to show their full therapeutic benefits in reducing inflammation and AS symptoms [17]. Emerging biological therapeutics focus on the inflammatory mechanisms that underlie AS, which may favorably affect the course of the disease and relieve symptoms, but not all AS patients necessarily need biological therapy. The decision to pursue biological therapy should be customized based on considerations such as elevated C-reactive protein (CRP) levels, which indicate increased disease activity, Smoking, which aggravates inflammation and raises the risk of severe symptoms, spinal involvement in AS, responsiveness to conventional medications, and the presence of symptoms that adversely influence the quality of life [17].

Diagnostic challenges and classification of ankylosing spondylitis

A chronic inflammatory disease that mainly influences the spine and sacroiliac joints and causes inflammation, AS causes pain and stiffness that worsens with time. AS is challenging to diagnose as its early symptoms can be confused with other illnesses; psoriatic arthritis, rheumatoid arthritis, and nr-axSpA are examples of inflammatory illnesses. Radiographic data, the primary basis for the diagnosis, supports the clinical presentation. AS can be divided into two categories: nr-axSpA, which is characterized by inflammatory involvement without radiographic evidence, and radiographic axial spondylitis, which is defined by evident sacroiliitis on X-rays. Both types of spondylitis are grouped under the general term axial spondyloarthritis [15,16]. In light of this, it is recommended to decide whether non-radiographic spondyloarthritis should be recognized as a separate condition or as a preclinical stage of AS. On X-rays, sacroiliitis is the characteristic radiographic feature that is visible, but using MRI enables the detection of early inflammatory changes before they show up on plain radiographs. Additionally, although the HLA-B27 gene is not unique to AS and is present in a sizable part of the general population, its presence can help confirm the diagnosis. Spinal radiography in AS showed a central radiodense streak corresponding to the dagger sign [18].

Inflammatory indicators and diagnosis in ankylosing spondylitis

Currently, erythrocyte sedimentation rate (ESR) and CRP level are the two non-specific inflammatory indicators that are commonly used to monitor the disease activity of rheumatic illnesses such as AS [19]. The heel’s back may occasionally also be affected. The hip and shoulder joints and the cartilage between the ribs and the breastbone may also be implicated. Inflammatory indicators such as CRP and ESR may also be increased in laboratory tests. Each person’s diagnosis of AS may or may not include normal acute-phase reactants (APRs) (such as CRP and ESR) [18]. APRs are markers of inflammation that are frequently elevated in inflammatory illnesses such as AS. However, the precise amount of APRs that increase varies among patients. When the disease first develops or when there is little disease activity, not all people with AS have high APRs [18,19]. As most cases of AS are effectively stopped for several years, and patients can resume their jobs, AS treatment involving X-ray therapy is a life-saving procedure [20].

Diagnosis and diagnostic criteria for ankylosing spondylitis

One example of a chronic inflammatory disease is AS, which primarily affects the spine and sacroiliac joints, causing pain and gradually worsening rigidity. As the symptoms of AS frequently coexist with those of nr-axSpA, inflammatory bowel disease, psoriatic arthritis, and reactive arthritis, diagnosing AS can be difficult, especially in the early stages. The patient’s clinical presentation serves as the primary criterion for diagnosis. However, supporting information from radiographic findings is also essential. Sacroiliitis, or joint inflammation in the sacroiliac joints, is a defining characteristic of AS, as shown on an X-ray. An X-ray is a crucial diagnostic technique for separating AS from diffuse idiopathic skeletal hyperostosis (DISH) [20]. Bilateral sacroiliitis, a stiff “bamboo spine” from vertebral fusion, and distinctive syndesmophytes are all visible on X-rays in AS patients.

On the other hand, DISH is distinguished by ossification that flows along the ligament in the anterior-longitudinal region, preserving the spine’s normal curvature and sparing sacroiliac joints [20,21]. Furthermore, even before they are discernible on standard radiographs, inflammatory alterations can be found early using MRI. The presence of the HLA-B27 gene is another diagnostic clue. AS is a genetic condition influenced by a complicated interaction between multiple alleles, including HLA-B27 and other HLA alleles (such as HLA-B40 and HLA-B41), as well as non-HLA (endoplasmic reticulum aminopeptidase one and interleukin-23 receptor) genetic variables [18].

Diagnostic considerations and imaging modalities in ankylosing spondylitis

Regional variations influence the prevalence of specific markers. It is important to remember that while this gene is linked to AS, it is also widespread in the general population, proving that it is not just a trait of people with AS. Laboratory tests may also show raised inflammatory markers such as CRP and ESR [14]. However, these are general signs. A thorough clinical examination, a complete medical history, and, most importantly, ruling out other possible reasons are necessary for diagnosing AS. Consideration should be given to MRI in cases where the history supports a diagnosis but plain radiography yields average results. MRI is beneficial in identifying early inflammatory lesions, bone marrow edema, sclerosis, fatty lesions, and enthesitis, especially at the sacroiliac joints [17]. It is also known for its sensitivity, which makes it the most effective imaging modality for identifying symptoms such as stiffness in the morning, inflammatory back pain, and possible involvement of peripheral joints [21]. It typically impacts the sacroiliac joints and spine, creating a bamboo spine. AS has a wide range of prognoses and courses. It is also linked to several related conditions, such as ulcerative colitis, psoriasis, Crohn’s disease, and uveitis (frequently associated with Reiter’s syndrome) [22]. Developed by Dr. Wojciech Maksymowych, the Maksymowych criteria offer a thorough framework for MRI-based assessment of structural and dynamic alterations in sacroiliitis. This framework has become essential to diagnose AS and other spondyloarthropathies.

To provide a comprehensive assessment of sacroiliac joint involvement, the Maksymowych criteria for diagnosing sacroiliitis using MRI incorporate both active inflammation and structural damage [17]. With precise definitions and scoring rules, the criteria provide consistency and guarantee consistent reporting and interpretation between readers for improved diagnostic accuracy [21]. Incorporating quantitative grading for specific observations allows for an unbiased evaluation of the severity of sacroiliitis, which aids in disease staging and well-informed treatment choices. High signal intensity on fat-suppressed T2-weighted imaging and gadolinium-enhanced sequences, indicating active inflammation, are characteristics of subchondral bone marrow edema in sacroiliitis [14]. Erosions indicate a persistent inflammatory injury and are well-defined abnormalities at the bone-cartilage interface on T1-weighted sequences with fat suppression. Thickness and augmentation of subchondral cortical bone on gadolinium-enhanced sequences, suggestive of early bone repair due to persistent inflammation, are symptomatic of cortical osteoarthritis [17]. Increased signal intensity and blurring of subchondral bone marrow on T1-weighted images, which represent chronic inflammation-induced bone remodeling and repair, are characteristics of subchondral sclerosis [21].

Infectious triggers and evolving understanding of reactive arthritis

While traditionally linked to AS and Reiter’s disease, bacterial respiratory infections are becoming more common (*Staphylococcus aureus*, *Streptococcus pneumoniae*, *Chlamydia psittaci*, *Streptococcus pneumoniae*), and viruses (COVID-19) have been linked to some cases of reactive arthritis. It is important to note that reactive arthritis now refers to a distinct entity related to infections, such as those caused by specific bacteria such as *Salmonella *or *Chlamydia *[22]. Unexpected changes in these symptoms can cause them to improve, worsen, or remain the same. The most frequently damaged bones are the lower back vertebrae, where ligaments and tendons link to bones, mainly the spine, and the joint where the base of the spine meets the pelvis. To accurately diagnose AS, extra-articular symptoms such as osteoporosis and fibromyalgia must be addressed. This can be done by a thorough clinical examination, recording a complete medical history, and methodically ruling out other possible causes. The management or treatment of AS is shown in Table 2 [21,22].

**Table 2:** Management or treatment of ankylosing spondylitis.

Treatment/Management category	Specific methods	Notes
Medications	Disease-modifying anti-rheumatic drugs	In ankylosing spondylitis, methotrexate and sulfasalazine improve peripheral joint involvement while reducing inflammation, symptomatic relief, and disease progression hindrance
	Tumor necrosis factor blockers	Biologics such as infliximab, etanercept, and adalimumab can reduce pain, stiffness, and disease progression
	Interleukin 17-A antagonist	Secukinumab and ixekizumab are examples. Reduces inflammation and associated symptoms
	Corticosteroids	It can be injected directly into joints or taken orally
Physical therapy	Exercises and stretching	It helps maintain flexibility, posture, and lung capacity. Regular exercising is essential
	Breathing exercises	This is especially important if the disease affects the ribcage
Surgery	Hip replacement	Considered if hip joints are damaged
	Spinal surgery	Rarely needed; only in severe cases or if the spine becomes unstable
Lifestyle and home remedies	Maintaining good posture	It helps reduce spinal deformity
	Applying heat or cold	It helps alleviate pain and muscle tension
	Sleeping on a firm mattress	Lying flat on the back without a pillow can prevent spinal curvature
	Regular exercise	Swimming is particularly beneficial as it exercises many joints without straining the spine
Alternative therapies	Acupuncture	Some people find relief through acupuncture, although scientific evidence is limited
	Massage	It can help with pain and muscle tightness, but the therapist should be knowledgeable about AS

Morning stiffness and hip and lower back pain are among the early signs of AS. The condition is characterized by tiredness and back discomfort that can extend to neck and hip pain, which is frequently unilateral or alternating [23]. Many treatments are available to manage the signs and symptoms of AS, even though there is no known cure for the condition. While NSAIDs such as ibuprofen and naproxen reduce pain and inflammation, regular exercise counteracts pain and reduces disease progression. DMARDs, such as more recent biologics, reduce joint edema and regulate the immune system to minimize inflammation [24,25]. Injectable corticosteroids provide temporary pain relief, and, in rare circumstances, surgery, such as kyphoplasty or joint replacement, may be advised to address significant joint injury or spine curvature.

Treatment strategies for ankylosing spondylitis

Moreover, most AS sufferers need lifetime medication (with potential side effects) to regulate their symptoms [26]. There are several ways to treat AS. NSAIDs, DMARDs, such as methotrexate, sulfasalazine, hydroxychloroquine, leflunomide, azathioprine, cyclosporine, tacrolimus biologics such as TNF blockers, and IL17-A antagonists are used as medications to treat pain and inflammation. Maintaining flexibility and lung capacity requires physical therapy, emphasizing stretches and exercises [17]. Severe cases may also require spinal surgery or hip replacements. It is advised to make lifestyle changes, including maintaining excellent posture, treating pain with heat or cold, resting on a firm mattress, and engaging in regular activities such as swimming. Some people also use complementary treatments such as acupuncture and massage, but the effectiveness and methodology should be carefully reviewed. The primary signs and symptoms of AS are stiffness and discomfort. Multiple goals of AS treatment include symptom relief, maintaining spinal flexibility, and avoiding consequences [20]. NSAIDs, which assist in reducing pain and inflammation, are frequently used as the first line of treatment. It also does not detail the specific clinometric used, such as Ankylosing Spondylitis Disease Activity Score (ASDAS) and Bath Ankylosing Spondylitis Disease Activity Index (BASDAI), or discuss how well the treat-to-target strategy works for AS. However, it does discuss the possibility of using biological drugs, such as TNF inhibitors, as a last resort when NSAIDs do not work or are poorly tolerated [21]. Other medications, such as TNF inhibitors, may be used when NSAIDs are ineffective or poorly tolerated. These minimize inflammation by concentrating on particular immune system proteins. The efficacy and safety of various drugs for AS treatment are described in Table 3.

**Table 3:** The efficacy and safety of various drugs for ankylosing spondylitis treatment. NSAIDs: non-steroidal anti-inflammatory drugs; TNF inhibitors: tumor necrosis factor inhibitors; IL-17 inhibitors: interleukin 17 inhibitors; DMARDs: disease-modifying anti-rheumatic drugs; COX-2 inhibitors: cyclooxygenase-2 inhibitors

Drug class	Drug examples	Efficacy	Safety	Notes
NSAIDs	Ibuprofen, naproxen	Effectively reduces pain and inflammation	Generally well-tolerated, but long-term use may lead to gastrointestinal issues or cardiovascular risks	It is not a disease-modifying therapy, but it is best for short-term relief
TNF inhibitors	Etanercept (Enbrel), adalimumab (Humira), infliximab (Remicade)	Rapid relief of symptoms, and improved function and quality of life	Common side effects include injection-site reactions, upper respiratory infections, and headaches	It can be expensive and require regular injections
IL-17 inhibitors	Secukinumab (Cosentyx), ixekizumab (Taltz)	Improves symptoms, spinal mobility, and quality of life	This may increase the risk of upper respiratory infections and gastrointestinal issues	A newer class of drugs with fewer side effects than TNF inhibitors.
DMARDs	Methotrexate, sulfasalazine	It provides relief and slows the progression of AS	Regular monitoring is required due to potential liver and blood-related side effects	It can take several weeks to months to work, which may not be effective for everyone
COX-2 inhibitors	Celecoxib	Offers pain relief with fewer gastrointestinal side effects	It can increase the risk of cardiovascular events, so should be used with caution in individuals with cardiovascular diseases	It is disease-modifying, but it is probably best for short-term relief

Ankylosing spondylitis disease activity assessment tools

ASDAS and BASDAI are essential tools in the assessment of disease activity in AS. These instruments aid healthcare professionals in evaluating disease severity, tracking its progression, and determining the effectiveness of treatment interventions. ASDAS is a composite index designed to assess disease activity in AS comprehensively. It considers various clinical and laboratory parameters, including patient global assessment of disease activity, back pain, peripheral joint pain or swelling, APRs (such as CRP or ESR), and duration of morning stiffness. By employing a specific formula incorporating these components, ASDAS generates a numerical score. Higher scores indicate increased disease activity, and the results can be categorized into different levels, such as inactive disease, low disease activity, moderate disease activity, and high disease activity.

On the other hand, BASDAI is a self-reported questionnaire that focuses on the patient’s subjective experience of symptoms over the past week. It includes questions about fatigue, spinal pain, joint pain, enthesitis (inflammation at sites where tendons or ligaments attach to bone), and morning stiffness. Patients rate the severity of each symptom on a scale from 0 to 10, and the overall BASDAI score is calculated as the mean of these individual scores. A higher BASDAI score indicates more significant disease activity, and it is often used alongside other clinical and laboratory assessments for a comprehensive understanding of the patient’s condition. ASDAS and BASDAI play crucial roles in clinical practice, aiding healthcare professionals in tailoring treatment plans to individual patient needs and monitoring treatment responses over time. These tools contribute to a holistic approach to managing AS, ensuring more effective and personalized care for individuals with inflammatory arthritis. AS disease activity assessment tools are presented in Table 4.

**Table 4:** Ankylosing spondylitis disease activity assessment tools.

Feature	ASDAS (Ankylosing Spondylitis Disease Activity Score)	BASDAI (Bath Ankylosing Spondylitis Disease Activity Index)
Type	Composite index	Self-reported questionnaire
Focus	Comprehensive assessment of disease activity	Patient’s subjective experience of symptoms
Components	Patient global assessment. Back pain. Peripheral joint pain/swelling. Acute-phase reactants (C-reactive protein, erythrocyte sedimentation rate). Morning stiffness duration	Fatigue. Spinal pain. Joint pain. Enthesitis. Morning stiffness
Scoring	Formula calculation with weighted values for each component. Scores are categorized into activity levels (inactive, low, moderate, high)	Individual item scores from 0 to 10. The overall score is the mean of all item scores
Interpretation	Higher score = increased disease activity	Higher score = more significant symptoms
Strengths	Objective and comprehensive. Includes laboratory data	Focuses on the patient’s perspective. Easy to administer
Weaknesses	More complex to calculate. Relies on self-reported data (patient global)	Less objective than ASDAS
Clinical role	Evaluate disease severity and progression. Monitor treatment response. Guide treatment decisions	Understand the patient’s experience. Track symptom changes
Overall	Provides a holistic assessment of AS	Contributes to patient-centered care

Multifaceted approaches in ankylosing spondylitis management

In people with AS, exercise reduces disease activity and function and has other advantages, such as preventing osteoporosis, improving respiratory function, and lowering cardiovascular risk. It may increase physical function and reduce disease activity, which improves general well-being. These effects are connected to measurements such as the BASDAI and the Bath Ankylosing Spondylitis Functional Index (BASFI). Surgery, such as joint replacements or spinal surgery, may be required in extreme circumstances. To control and slow the progression of the disease, patients are frequently urged to make lifestyle changes, such as improving their posture, sleeping on a firm mattress, and giving up smoking. Chronic inflammatory disease (AS) that affects the spinal column and sacroiliac joint is characterized by elevated expression of HLA-B27 [27]. While bone marrow edema is not exclusive to AS, it is vital to remember that it can be a distinguishing feature of inflammatory arthritis. Differentiating characteristics may include the precise location and patterns observed, as bone marrow edema can be seen in various pathologies.

Additionally, if bone marrow edema is seen in a distinctive subchondral bone on an MRI of the sacroiliac joints, it indicates spondyloarthritis [28]. While evidence about golimumab survival rates in AS patients should be assessed, it is crucial to remember that etanercept has demonstrated clinical effectiveness and security in patients with active AS that has lasted for up to seven years of follow-up [29]. For AS, inpatient spa exercise therapy combined with group physiotherapy is more beneficial than group physiotherapy. Personal training regimens at home or under supervision are better than no intervention [30]. In addition, physical therapy, NSAIDs, DMARDs, and corticosteroids are frequently added in certain situations [17,24,25].

Table 5 presents a summary of the included studies.

**Table 5:** A summary of included studies. AS: ankylosing spondylitis; IL-23: interleukin 23; IL-17: interleukin 17

Author(s)	Year	Main characteristics
Garcia-Montoya et al. [1]	2018	Recent developments in our knowledge of AS, with an emphasis on its comprehension and treatment
Golder et al. [2]	2013	An updated overview of AS providing current information on the condition
Fiorillo et al. [3]	2019	Explores AS and its associations with immune-mediated disorders, broadening the understanding of the disease
Hanson et al. [4]	2017	Investigates the genetic factors and causes contributing to AS
Zhang et al. [5]	2021	Examines the multi-target mechanism of Tripterygium wilfordii Hook for AS treatment, utilizing network pharmacology and molecular docking approaches
Chen et al. [6]	2021	Developments in the pathophysiology, causation, and treatment of inflammatory bowel disease
Baeten et al. [7]	2020	Examination of the reasons behind the lack of efficacy in IL-23 inhibition for AS
McGonagle et al. [8]	2021	Examination of the reasons behind the ineffectiveness of IL-23 inhibition on AS
Martins et al. [9]	2014	Exercise and AS using the modified New York criteria. A systematic review of controlled trials with meta-analysis
van der Heijde et al. [10]	2019	The influence of treatment on the structural development of AS is evaluated using the Modified Stoke AS Spinal Score as an outcome measure
Dubash et al. [11]	2019	Investigating IL-17A blockage in AS: secukinumab, ixekizumab, and further options
Souza et al. [12]	2017	A randomized controlled study showed that walking performance and muscle strength improved with Swiss ball workouts in patients with AS
Mitsui [13]	2008	Overview of AS published in Clin Calcium in 2008
Crossfield et al. [14]	2021	Examination of shifts in the incidence, prevalence, and diagnostic time of AS throughout a 20-year period
Slobodin et al. [15]	2012	AS is considered a field in progress, explored in the Israel Medical Association Journal in 2012
Braun et al. [16]	2018	Novel characteristics and differential diagnoses related to imaging of axial spondyloarthritis
Sieper et al. [17]	2002	A review of AS was released in the 2002 Annals of Rheumatic Diseases
Mukai et al. [18]	2022	Examination of the “dagger sign” in AS, published in Internal Medicine Tokyo in 2022
Wu et al. [19]	2021	The correlation between disease activity and the systemic immune inflammation index in individuals suffering from AS
Sambrook [20]	1972	An overview of AS published in the British Medical Journal in 1972
Maksymowych [21]	2004	AS discussed as “not just another pain in the back” in an article published in the Canadian Family Physician in 2004
Woodward et al. [22]	2009	Recent developments and anesthetic implications of AS explored in Anaesthesia in 2009
Mayo Clinic [23]	2023	Mayo Clinic’s overview of AS symptoms and causes, accessed in 2023
Cleveland Clinic [24]	2020	Cleveland Clinic’s information on AS symptoms, causes, and treatment, accessed in 2023
Thomas et al. [25]	2010	Genomics of AS explored in Discovery Medicine in 2010
Nancy et al. [26]	2021	AS genetics research that led to novel biology and the identification of potential treatment targets was published in Frontiers in Immunology in 2021
Suh et al. [27]	2017	A middle-aged woman’s case of AS linked to primary aldosteronism was covered in the Korean Journal of Internal Medicine in 2017
Poddubnyy [28]	2020	Examination of the challenge of diagnosing axial spondyloarthritis: classification vs. diagnostic criteria, published in Rheumatology in 2020
Sari et al. [29]	2015	2015 saw a discussion on the management of AS in the Turkish Journal of Medical Sciences
Dagfinrud et al. [30]	2008	The Cochrane Database of Systematic Reviews evaluated physiotherapy therapies for AS in 2008

## Conclusions

An excellent illustration of the complex interplay among genetics, immunology, and environment is AS. Since Galen first described AS, it has significantly impacted the medical world due to its impact on the axial skeleton and sacroiliac joints. It is admirable how far medicine has come from the 19th century, focusing on precise diagnosis, to the present. A paradigm shift in how we think about the genetic roots of the disease has been brought about by our modern understanding of over 100 genes, with the HLA-B27 variation playing a leading role. The development of biological therapies, driven by a profound understanding of inflammatory pathways, has ushered in a new age for AS patients. These medications, which range from NSAIDs to TNF-specific therapy, have slowed the progression of this crippling illness while also reducing symptoms. Numerous people have optimism thanks to numerous therapeutic approaches, physical therapy, and lifestyle changes. It is important to understand that AS still presents several obstacles. Research into topics such as the IL-23 pathway and its wider ramifications is crucial because several nuances of the illness are still not fully understood. The relationship between AS and other disorders, including Reiter’s syndrome and Crohn’s disease, denotes a broader range of issues that require coordinated care. In conclusion, even though we have made significant progress in understanding, diagnosing, and treating AS, we still have a long way to go. The continuing collaboration of genetic research, pharmacological development, and comprehensive patient care continues to be our best action toward eradicating this disorder.
